# The short Persian version of motorcycle riding behavior questionnaire and its interchangeability with the full version

**DOI:** 10.1371/journal.pone.0201946

**Published:** 2018-08-30

**Authors:** Hojjat Hosseinpourfeizi, Homayoun Sadeghi-Bazargani, Kamal Hassanzadeh, Shaker Salarilak, Leili Abedi, Shahryar Behzad Basirat, Hossein Mashhadi Abdolahi, Davoud Khorasani-Zavareh

**Affiliations:** 1 Department of Orthopedics, Tabriz University of Medical Sciences, Tabriz, Iran; 2 Road Traffic Injury Research Center, Tabriz University of Medical Sciences, Tabriz, Iran; 3 Department of Statistics &Epidemiology, Tabriz University of Medical Sciences, Tabriz, Iran; 4 Department of Public Health, Islamic Azad University of Tabriz, Medical School, Tabriz, Iran; 5 Department of Epidemiology, Kerman University of Medical Sciences, Kerman, Iran; 6 Traffic Police Applied Research Center, Tehran, Iran; 7 Tabriz Health Services Management Research Centre, Tabriz University of Medical Sciences, Tabriz, Iran; 8 Safety Promotion and Injury Prevention Research Center, Tehran, Iran; 9 School of Health, Safety and Environment, Shahid Beheshti University of Medical Sciences, Tehran, Iran; Qazvin University of Medical Sciences, ISLAMIC REPUBLIC OF IRAN

## Abstract

The aim of current study was to develop a valid and reliable short motorcycle riding behavior questionnaire (SMRBQ) and assess its interchangeability with the original 48-item tool. Through a psychometric study in Bukan district of Iran in 2015, the most recent available Persian version of the motorcycle riding behavior questionnaire (MRBQ) was used as a reference to develop its short version, the SMRBQ. The preliminary version was prepared through expert reviews, and its dimension was further reduced through principal component analysis (PCA). An exploratory factor analysis (EFA) was then applied on the remaining items and the final version was developed with 23 items. The validity, consistency, agreement and interchangeability of the SMRBQ were assessed in parallel with the MRBQ using several statistical methods including Kendall’s Tau, intra-class correlation coefficient (ICC), Bland-Altman plot and receiver operating characteristics (ROC) curve analysis. All the 340 participants were males. The mean age of the participants was 30.2 (SD = 9.1). SMRBQ was developed including 23 items. The mean normalized score for the full version was 30.5 (SD = 11.2) and it was 30 (SD = 13.9) for the short version. There was a high correlation between the normalized scores of MRBQ and SMRBQ (Kendall’s Tau = 0.82). The ICC of the interchangeability of the full version and short version scales was as high as 0.92 (95% CI: 90.2–93.5). The scale had adequate internal consistency based on the calculated Cronbach’s alpha which was 0.85 for the scale. Bland-Altman and ROC curve analysis confirmed the interchangeability and criterion validity of the SMRBQ. The Persian version of SMRBQ was found to be a valid, reliable and feasible tool for assessing motorcycle riding behavior in the studied population.

## Introduction

Motorcycle users are part of the road users with higher vulnerability to traffic injuries. Half of the people dying on the world’s roads are pedestrians, cyclists and motorcyclists known as “vulnerable road users”[1]. Nearly one-fourth of all the road traffic fatalities belong to motorcyclists. The proportion of motorcyclist fatalities is largely unchanged worldwide or even increased in some regions since 2010 [1]. Considering the lower passive and active safety specifications in motorcycles compared to 4-wheel vehicles as well as the individual characteristics of motorcycle riders versus other vehicle drivers, the role of riding behavior is much more pronounced among motorcycle riders [2–4]. Investigating the riding behaviors of motorcyclists and assessing risky riding behaviors is essential for planning the motorcycle safety promotion interventions. Several studies have been done to develop tools for assessing motorcycle riding behaviors [5–7]. In order to investigate motorcycle riding behavior it should be taken into account that motorcycle riders comprise a specific subpopulation of vehicle users with various purposes for using motorbikes other than just transportation. These might include such purposes as: recreation, sporting, delivery of goods, carrying passengers and even illegal activities [8–13]. Using lengthy questionnaires, regardless of their benefits, has its own limitations such as being costly and time-consuming. It can also lead to lower participation in some circumstances. Developing short versions of the available tools with acceptable interchangeability could be of high value in conducting studies when there is a shortage in time or financial support or when there is a risk of unacceptable attrition if the data collection tools are long enough to affect participation. The current Persian motorcycle riding behavior questionnaire has 48 items. In order to be able to conduct roadside surveys, completing a 48-item questionnaire along with other background information or complementary tools affect the feasibility of conduct especially among the young motorcyclists. This may affect both the attrition and completeness of information. Validated short tools for assessing motorcycle riding behavior are sorely available and no such a tool exists in Persian. The aim of this study was to develop a valid and reliable short motorcycle riding behavior questionnaire (SMRBQ) in Persian and assess its interchangeability with the original 48-item motorcycle riding behavior questionnaire (MRBQ).

## Methods

This psychometric study was done through a cross-sectional design on 340 motorcycle riders of Bukan district of Iran in 2015. Bukan is the capital of Bukan County, West Azerbaijan Province, Iran. As of 2017, its population was estimated to be about 195,000 people living in 57,000 families. The city is a mountainous area and its distance from the provincial capital, Urmia, is 184 KMs. Seventy-five percent of the population is settled in the urban places and 25% are living in the rural areas. Bukan is reported to have 25% of the whole traffic crashes in the province with above 90 annual traffic mortalities, which is 1.5 times higher than the national rate [14]. A cluster random sampling method was applied to enroll the participants. The entire city was divided into 14 clusters based on the geographic areas covered by urban health centers. Then, 7 clusters were randomly selected out of these 14 clusters. By referring to motorcycle repair shops and the homes and workplaces of the motorcycle riders in each cluster, the data were collected. The inclusion criteria for the participants were: riding a motorcycle at least three times in a month, being over 15 years of age, and willingness and capability to complete the questionnaire. The sample size was estimated for a final measurement of behavior and its predictors through a master thesis study as the base survey. The sample size was calculated using Stata to principles of cluster sampling method.v11 Sampsi based on parameters extracted from the study by Abedi et al.. Having the standard deviation of 22.96, the confidence level of 95% and accuracy of 3, a total of 227 people were estimated for initial sample size. According to principles ofcluster sampling method, the estimated number was multiplied by a design effect coefficient of 1.5. The final sample size included 340 people. This amount satisfies also the rules of thumb for factor analysis as well as the correlation-based sample size estimation using Fisher’s Z-transformation.

Other than the demographic data, a modified full version of MRBQ was discussed in an expert panel of traffic injury researchers and language experts to assess the quality and appropriateness of the translation for the only available Persian MRBQ which was previously validated by Motevalliyan et al. in 2009 upon translation and modifications on the MRBQ [15–17]. The MRBQ was used as the reference to develop the short version had 48 items. As the short version was an extraction of the full version along with four more merged new questions, the participants completed a questionnaire with 52 riding behavior items. These items were later used in statistical processing to develop and assess interchangeability of the short version with the full version. In this questionnaire, the answers for each item of the questionnaire have five Likert-scaled choices as "never = 0", "seldom = 1", "sometimes = 2", "often = 3" and "always = 4". Only a modification in item 23 of the Persian questionnaire was applied as follows: the term “pull away” (move off/move ahead) was mistakenly translated as “pull off” (to leave the main roadway).

In order to prepare the short version of MRBQ, the full version of the questionnaire was sent out to ten experts in the field of road traffic injury prevention including injury epidemiologists, public health researchers, police officers with research expertise, and municipality traffic expert officers. They were selected from the National Road Traffic Knowledge Development Trustee’s database of experts (now available as SafeLir.com). They were requested to prioritize the MRBQ items according to their importance and select 12 of the 48 items from the full MRBQ that they thought were more relevant and necessary to be included in a short version of the questionnaire. Although in preparing short versions of questionnaires both expert reviews and statistical methods could be used, a combined methodology could be useful to take advantage of theoretical concepts along with patterns of the correlations among the items [18]. When several experts select 25% of the items, frequency of the selections for each items is an indication of the priority of the item. Another method could be to ask raters to score all the items. As the purpose of current study was to develop a short version, we preferred the frequency-based priority setting.

Those items that were not selected by none of the ten experts were removed. For items that were suggested only by one expert, the decision to keep or remove the item was made on later statistical process including PCA and EFA. Afterwards, the remaining items were entered into a dimension reduction statistical process using PCA to remove the items which showed low contribution in PCA. Variables not correlated with any principal component or correlated with the last dimensions were considered as the variables with low contribution and considered for removal through dimension reduction [19]. An EFA was applied on the remaining items. The EFA approach was done using principal factor extraction with Varimax rotation. The reason behind using an orthogonal rotation (varimax) was assuming that various types of riding behaviors (such as those usually classified as error-type items vs. violation-type items) are not essentially considered to be correlated. The number of included factors was decided upon assessing slope change in scree plot with a conditional lowest level of eigenvalue equal to one. Uniqueness less than 0.7 were the tentative statistical criteria for selecting the items.

Kaiser–Meyer–Olkin (KMO) value was carried out to assess sample adequacy and sufficiency of the factor analysis model. An item level KMO value above 0.60, and total scale KMO minimum acceptable level was set at 0.7 [20]. Bartlett's test of sphericity was also applied to assess the sphericity expected to yield significant results in order to find the model acceptable. The extra conditions applied to assess the appropriateness of the factor analysis model included an item-total correlation index above 0.20, and factor loads above 0.40 in at least one factor.

In order to assess the reliability of the SMRBQ, the internal consistency of the final dimensionally reduced factor analysis model was assessed using Cronbach's alpha coefficient calculated for each factor as well as total scale separately.

1. Validity, consistency, agreement and interchangeability of the SMRBQ were assessed in parallel with the MRBQ using several statistical methods as follows:
2. Kendal’s Tau-b as for the consistency and agreement assessment purposes respectively. Pearson correlation coefficient, although not an appropriate single method for assessing agreements, could be benefitted as a complementary approach to assess how the scores of two methods covariate. However, Kendal’s Tau, as a rank correlation coefficient, could be used as a measure of agreement between parallel assessments [21].
3. Scatter plots of the normalized scores of SMRBQ and MRBQ for consistency assessment.
4. ICC for interchangeability assessment: ICC is a well-known method of assessing interchangeability. ICC was first presented by Fisher half a century ago as a modification of Pearson correlation coefficient. Recent version of the ICC, however, is calculated by mean squares obtained by analysis of variance. It is a reliability measure used to assess either degree of consistency or absolute agreement [22]. ICC is a more desirable measure of reliability that indicates both degree of correlation and agreement between measurements. Various forms of ICC could be calculated through different statistical methodologies. Ten forms of ICC in three groups have been defined by McGraw and Wong. The first group is defined based on Model types as; 1-way random effects; 2-way random effects; and 2-way mixed effects. In 1-way random effects model, which is rarely used in clinical reliability analysis, each subject is rated by a different set of raters randomly chosen from a larger population of possible raters. 2-way random effects model is mostly appropriate for evaluating rater-based clinical/health assessment methods and when planning to generalize the reliability. In 2-way mixed effects model reliability is not intended to be generalized. The second group is defined based on Type as; single rater/measurement and the mean of k raters/measurements. The third group is defined based on definition of the relationship considered to be important including consistency and absolute agreement. A combination of the elements in these groups gives rise to 10 forms of ICCs [23]. In this study, the absolute ICC was derived from both two-way random/mixed effects modeling for individual application. As the short version is planned to be used as an alternative, the individual (single) rater value was considered. As the normalized scores were used in this study, the absolute ICC was calculated.
5. Bland-Altman method for criterion validity assessment: As in this study the full version MRBQ score could be considered as the gold standard of comparison, the criterion validity could be assessed using Bland-Altman graphical approach but with a modification upon the original Bland-Altman method which uses the gold method score as abscissa values instead of traditional use of average value. The MRBQ normalized score was assigned as the X-axis value and the difference between scores of the two methods (i.e., the bias) was given as the Y-axis values [24–26].

Diagnostic value validity assessment: Considering the fact that those at higher scores of MRBQ are for criterion at higher risk of road traffic injuries, the capability of SMRBQ in detecting those who belong to the highest decile of MRBQ score was assessed using diagnostic value methods. The ROC curve was piloted along with reporting the area under ROC curve.

Normalized scores were used for scoring the SMRBQ values. In order to normalize the scores, a value from 0–100 was allocated to each item according to the formula; “Question score = (x-Min)*100/(Max-Min)” where x = preferred switch, Min = first switch number, Max = latter switch number. The total scores could be normalized similarly. The data were analyzed using Stata statistical software package version 13.1 (StataCorp, Texas). The study was approved by the Ethics Committee of Tabriz University of Medical Sciences. Moreover, for ethical considerations, informed consent was obtained from all the participants in this study.

A copy of the Short Motorcycle Riding Behavior Questionnaire (SMRBQ) in Persian with descriptions of the items in English is provided in S1 Table as a supplementary file.

## Results

**Fig 1:**
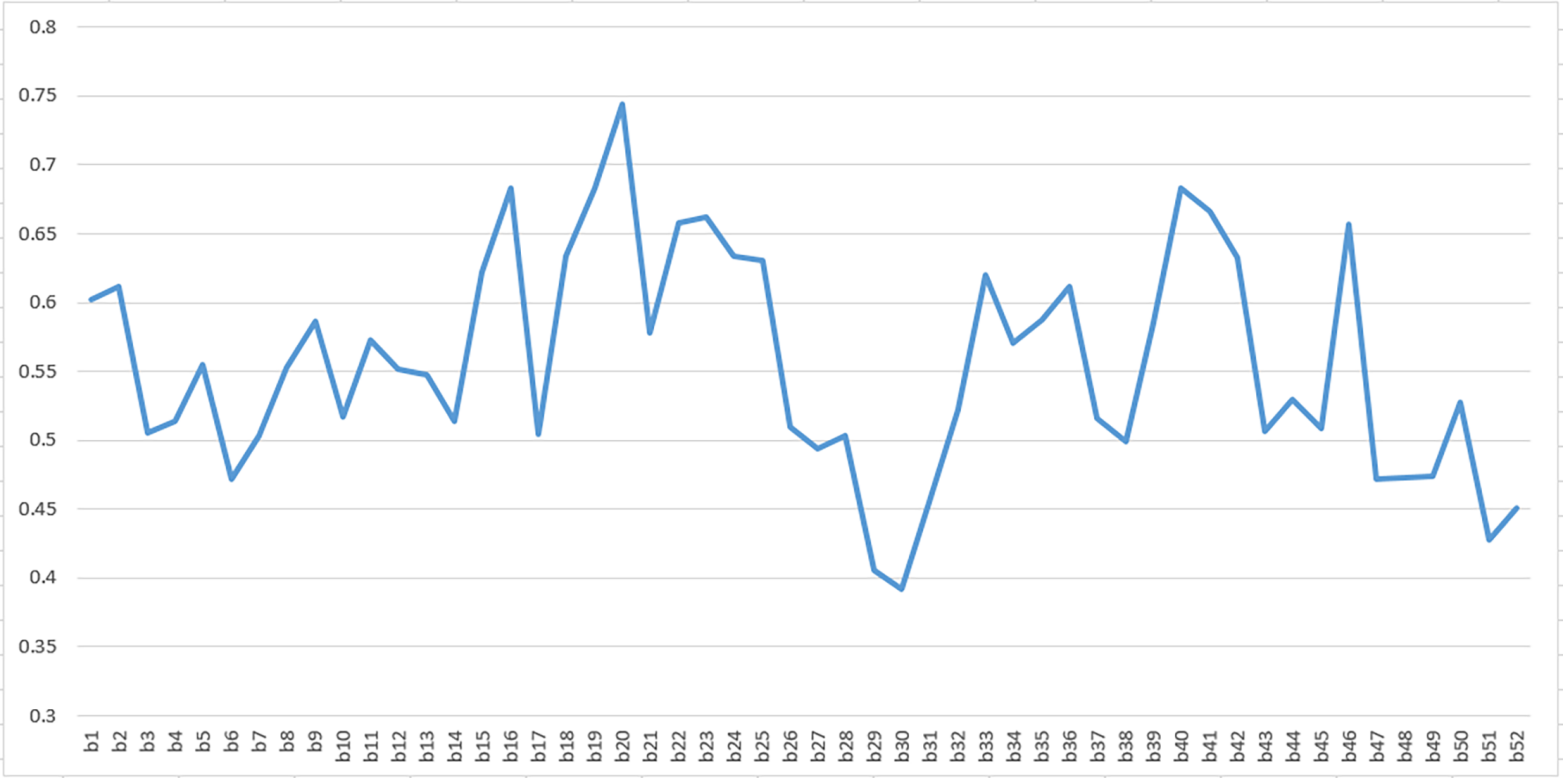
Squared multiple correlations of variables with all other variables derived from preliminary principal components analysis of all variables for assessing motorcycle rider behavior. Footnote: X-axis: Questionnaire items Y-axis: Squared multiple correlations of variables with all other variables.

All the 340 participants were males with a mean age of 30.2 (SD = 9.1). All the participants agreed to participate in the study after informing them about the study purpose and standards. Eight percent of the riders were illiterate, 32% had academic education and the remainder had primary or secondary education. Also, 194(57.1%) of the riders were married. With respect to the riding experience 271(79.7%) participants had above one year experience of riding motorcycle and only three of them being novice riders with an experience below one month of riding. Two-thirds of the participants used to ride motorcycle at least four days a week. Only 100(29.4%) of the riders had a riding license. The preliminary short version, based on the recommendations of experts, included 38 items. The results of preliminary PCA showed that some items such as items No. 29 & 30, dropped through expert comments, had also lower squared multiple correlations of variables with all other variables (Fig 1).

**Fig 2:**
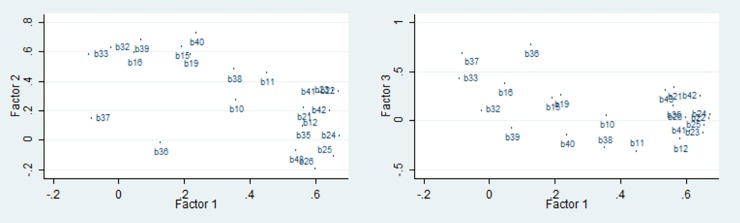
The loading plots of the first factor against second and third factors after exploratory factor analysis of the motorcycle riding behavior questionnaire using principal-component factor method and varimax rotation.

The dimension reduction based on the EFA ended in 23 items extracted in three factors as given in Table 1. The loading plot of the first factor against the second and third factors is given in Fig 2.

**Table 1:** List of the included items of the short motorcycle riding behavior questionnaire, their mean scores and the factor structure matrix.

*Items	Item description	Mean item score	Item-factor correlations		
			Factor 1	Factor 2	Factor 3
b10	Tailgating the vehicles in front	1.3	**0.359**		
b11	Wide ride going round the corners	0.82	**0.452**	0.451	
b12	Speeding (when reaching corners)	1	**0.584**		
b15	Speeding (motorways)	1.4		**0.634**	
b16	Speeding(residential roads)	1.4		**0.593**	0.3794
b19	Riding between fast lanes of traffic	1.2		**0.582**	
b21	Scaring speeding (when reaching corners)	1	**0.566**		0.3354
b22	Wheelie attempts	0.79	**0.673**	0.332	
b23	Off road due to very quick pull away	0.85	**0.655**	0.337	
b24	Wheel spin (on purpose)	0.94	**0.674**		
b25	Wheel spin (unintentional)	0.78	**0.657**		
b26	Riding at night just with dipped light	0.89	**0.602**		
b32	Carrying heavy weight	1.4		**0.626**	
b33	Ride with more than one pillion passenger	1.8		**0.580**	0.427
b35	Riding impaired motorbike	1.1	**0.562**		
b36	Not using helmets while riding	2			**0.7758**
b37	Not using helmets by pillion passengers	2			**0.6862**
b38	Riding while on drugs or medications affecting riding safety	1	**0.353**	0.480	
b39	Likely of hitting opened car doors	1.4		**0.680**	
b40	Passing the red lights	1.2		**0.728**	
b41	Riding against the legal traffic direction	1.1	**0.613**	0.324	
b42	Sidewalk riding	1.2	**0.646**		
b43	Mobile conversation or messaging while riding	1.1	**0.541**		0.309

Correlations <0.3 left blank

Bolded correlations represent the assigned factors

*: item numbers are based on original version for comparison reasons

The list of the included items of the scale, their mean scores as well as the structure matrix for correlations between variables and varimax rotated common factors are presented in Table 1. In addition, the average time to complete each short questionnaire was eight minutes.

**Fig 3:**
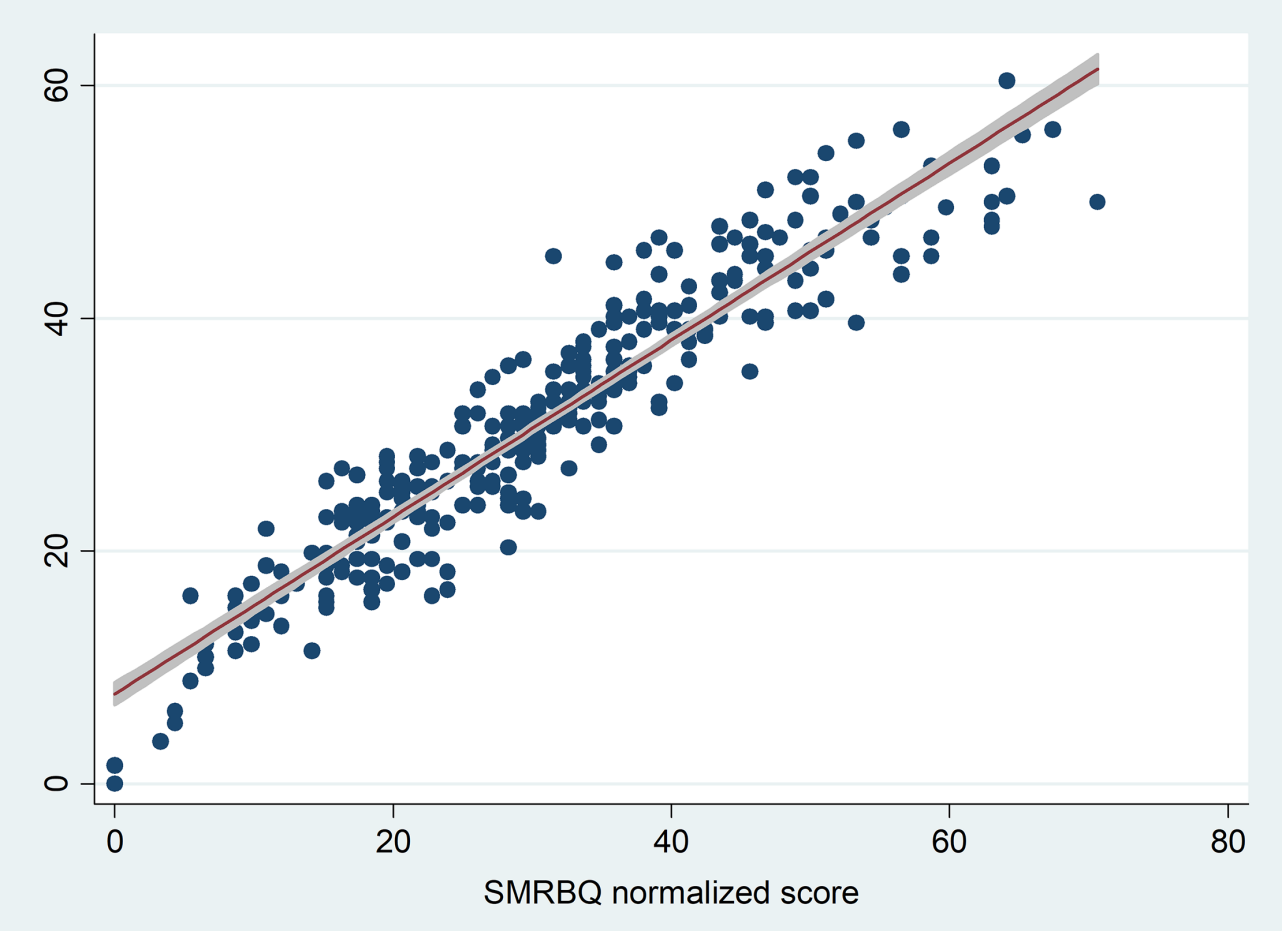
The scatter plot for assessing the linear correlation between the short and full versions of motorcycle riding behavior questionnaire. Footnote: Y-axis: MRBQ normalized score. MRBQ: Motorcycle riding behavior questionnaire SMRBQ: Short motorcycle riding behavior questionnaire. The red light is the fitted linear regression line with its 95% confidence interval shown as grey line shadow.

Mean normalized score for the full version was 30.5 (SD = 11.2) and the mean normalized score for the short version was 30 (SD = 13.9). There was a high correlation between normalized scores of MRBQ and SMRBQ (Fig 3). The Kendall’s Tau coefficient to assess the agreement between the two scales was 0.82 (P<0.001).

The absolute individual ICC of the interchangeability of the full version and short version scales was as high as 0.92 (95% CI: 90.2–93.5) equally for both mixed and random error two-way modeling.

**Fig 4:**
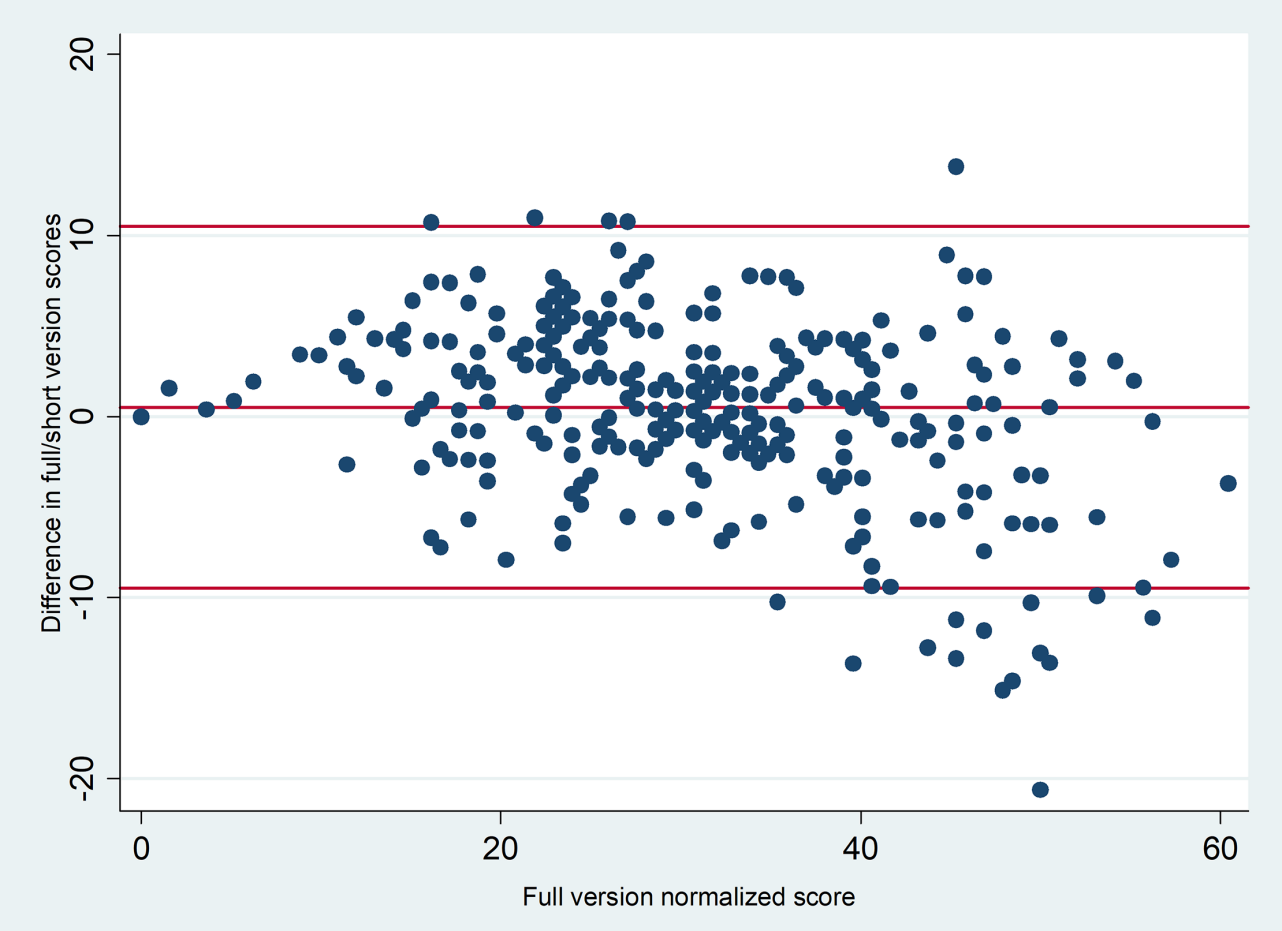
The Bland-Altman graph for assessment of the short and full versions of motorcycle riding behavior. Footnote: The middle red line represents the average bias Line. The bias is computed as the value determined by one method minus the value determined by the other method. The upper and lower red lines are the limits of agreement, computed as the mean bias plus or minus 1.96 times its standard deviation.

The Bland-Altman graph assessing the agreement between the SMRBQ and MRBQ over the range of normalized scores is given in Fig 4.

**Fig 5:**
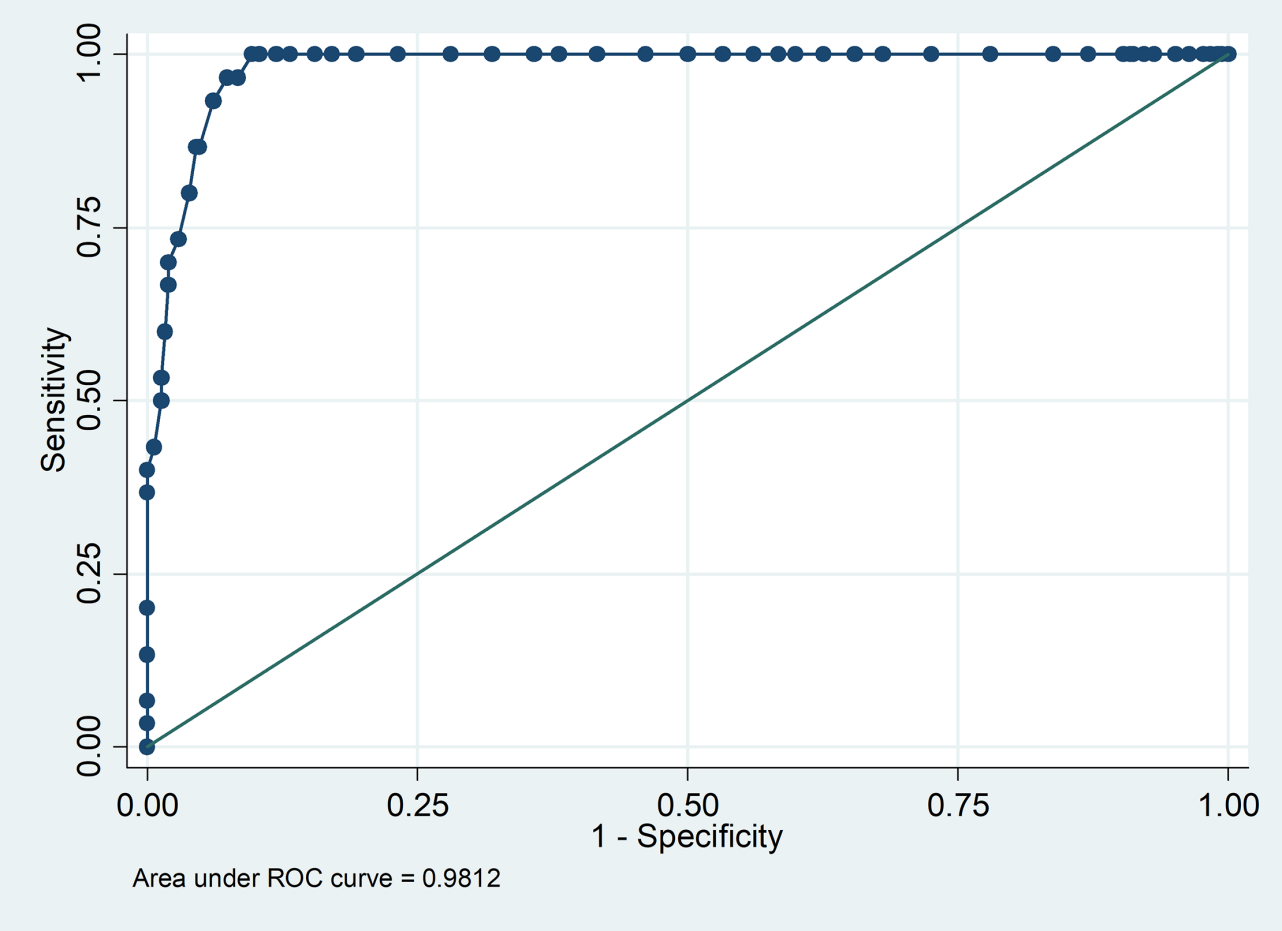
Receiver operating characteristics curve for SMRBQ score in detecting whether a rider is in the last decile of risky riding behavior score considering the MRBQ as gold standard.

ROC curve analysis confirmed the criterion validity of the SMRBQ. The SMRBQ had high diagnostic value in detecting those having a score at the last decile of MRBQ scores with 0.98 area under ROC curve and above 93% sensitivity and specificity with a SMRBQ cutoff normalized score of 46.7 (Fig 5).

The scale had adequate internal consistency based on the calculated Cronbach’s alpha which was 0.85 for the scale. Cronbach’s alpha was equal to 0.86 for the first factor subscale, 0.68 for the second factor and 0.69 for the last factor subscale. The three factors extracted could be labelled as follows:

1. Main factor: Unfit erroneous riding, intrusive and exhibitive behaviors including items 10,11,12, 21,22,23,24,25,26,35,38,41,42 & 43
2. Second factor: Time and money opportunistic behaviors including items 15,16,32,33,39 & 40
3. Third factor: Helmet use behaviors including items 36 & 37

All items categorized carried the highest correlation with the corresponding factor except for item 38 which was included in first factor due to its theoretical concordance.

## Discussion

The present study revealed that SMRBQ had adequate content and structural validity and internal consistency. Its scores were in high agreement with the scores of the full version MRBQ.

Very few scales have been developed for motorcycle riding behavior assessment. For instance, Chinese motorcycle rider driving violation questionnaire (CMRDVQ) assesses the driving violations of Chinese motorcycle riders and evaluates its screening accuracy between accident-involved and accident-free motorcycle riders. This scale consists of 19 items [7].

SMRBQ was found to have a three-factor solution with high internal consistency for the whole scale and main factor as well as acceptable internal consistency for the second and third factor subscales. The full version MRBQ has been reported to have 4 to 6 factors to explore the scale structure. However, CMRDVQ applied in a Chinese population which was a parsimonious scale was found to have a two-factor solution having a high internal consistency like SMRBQ [6, 7, 15, 17]. The items explored in CMRDVQ were mostly similar with SMRBQ including items such as riding against the traffic flow, passing the red light, speeding, side-walk riding, and riding too close to the front vehicle. In addition, CMRDVQ had included an item as “Sounded your horn to indicate your annoyance to another road user”. Such item was not included in our scale, because although it is a violation behavior, but it is not a safety issue for motorcycle riding. MRBQ has been assessed for factor validity in the United Kingdom and Turkey revealing the scale to have five factor structures [6, 15]. An Australian version has also been validated with 43 items and four factor structures, though merely among novice riders [5]. The item on deliberate annoyance by another rider, included by previously mentioned scales, was removed in present study on SMRBQ. Although this is considered as an emotional response in the aforementioned studies, it does not seem to be relevant for riding behavior scales because they are supposed to assess rider’s behavior not the occurrence of counterpart behaviors. Sakashita also found that this item is a clear form of cross-loading between two factors [5]. The only wearing-related items in SMRBQ were those related to wearing helmets; and wearing leather motorcycle suit was not included in SMRBQ because currently in the study setting in Iran or similar countries, no such a clothing is used by motorcyclists except for the sports riders, and no doubt, lack of variance through factor analysis leads to exclusion of the items. However, this item can be added in the future or if the scale is used in high-income countries. Other than pure lack of variance, rarity (while taken into account along with importance of the item) is another fact that could be considered in developing the short version. This is especially of importance for novice riders due to their lower age and experience which are considered as the risk factors of motorcycle traffic injuries [27–29]. We highly recommend the researchers using SMRBQ to collect some additional information potentially related with riding behaviors or accident risks such as having a valid riding license according to the local legislations, riding experience, purpose of riding, age and gender [13, 29–31].

Though it is nearly impossible to escape from small amounts of bias while shortening the questionnaires, the multi-method approach of assessing the validity showed that such a bias is small enough in trade off for the large gain in feasibility. Although, the nonzero intercept suggests existence of a small amount of bias, this had to be further examined through Bland-Altman graphical agreement assessment which showed that bias started to increase between SMRBQ and the full version of MRBQ over the abscissa decreasing the agreement between the scores at higher deciles and the short and full versions deviated more at higher scores. This pattern gave rise to the question that whether the short version could be found valid enough in detecting those with higher risky behavior scores or not? This was the reason for using ROC analysis that hopefully confirmed the validity of the short version with respect to such concern. The results of ROC curve analysis showed that SMRBQ score was highly reliable in detecting whether a rider is in the last decile of risky riding behavior score.

The present study provided a short feasible tool for valid and reliable assessment of motorcycle riding behavior in shorter time than for using long questionnaires. No doubt, using a full version questionnaire has many benefits especially for assessing the rare behaviors, but long questionnaires have also their own limitations such as being costly and time-consuming. The use of lengthy questionnaires in some circumstances may also lead to higher attrition rates and its subsequent effects. Such occasions are most likely for motorcycle rider populations especially when the samples are taken from the traffic environment affecting the riders travelling purposes. Commercial motorcycle riders are of higher importance in this regard, because they may not risk diminishing their income by putting time to answer an abundance of questions. Also, the use of SMRBQ, while losing very small proportion of information but saving above 50% of time, could be an alternative to using the full version MRBQ when there are time, cost and participation interest limitations.

## Conclusion

The Persian version of SMRBQ with 23 items was found to be a valid, reliable and feasible tool for assessing motorcycle riding behavior in the studied population and similar settings. It has acceptable interchangeability with the full version MRBQ and could be used as an alternative when there are limitations in using the 48-item full version MRBQ. Future research in other populations could improve assessing the external validity of SMRBQ other than the investigated population.

### Limitations

In the present study, we considered the full version as the gold standard for assessing the short version. The reason behind this was that the full version is considered as the source tool. However, when it comes to the outcome that a riding behavior questionnaire may predict, there is no guarantee for the full version questionnaire to have a better outcome prediction than the short one. The best way in evaluating riding /driving behavior tools could be to test them in cohort or case-control studies taking the incidence of injuries/crashes as the gold standard. But this could not be applied in present study. Although this study was a psychometric survey and its aim was not to investigate the behaviors in Bukan district and to extrapolate it to Iranians, it should be taken into account that even the psychometric results could suffer from generalizability limitations (e.g., applicability to females in our case). Although the test-retest reliability of the full version is reported earlier, not repeating the test-retest reliability of the short scale should be considered as a limitation for present study.

## Supporting information

S1 Data Data used for analysis in microsoft excels format. (XLS)

S1 Table Short Motorcycle Riding Behavior Questionnaire (SMRBQ): Questions in Persian with descriptions in English. (DOCX)
