# Correction: The short Persian version of motorcycle riding behavior questionnaire and its interchangeability with the full version

**DOI:** 10.1371/journal.pone.0211351

**Published:** 2019-01-23

**Authors:** Hojjat Hosseinpourfeizi, Homayoun Sadeghi-Bazargani, Kamal Hassanzadeh, Shaker Salarilak, Leili Abedi, Shahryar Behzad Basirat, Hossein Mashhadi Abdolahi, Davoud Khorasani-Zavareh

There are errors in affiliations 8 and 9 for author Davoud Khorasani-Zavareh. Affiliation 8 should be: Safety Promotion and Injury Prevention Research Center, Shahid Beheshti University of Medical Sciences, Tehran, Iran. Affiliation 9 should be: School of Public Health and Safety, Shahid Beheshti University of Medical Sciences, Tehran, Iran.
