# Supplementary material for: The short Persian version of motorcycle riding behavior questionnaire and its interchangeability with the full version
Source: PLoS One. 2018 Aug 30;13(8):e0201946. doi: 10.1371/journal.pone.0201946 (PMC6116927; doi:10.1371/journal.pone.0201946)
Supplement: S1 Table — (DOCX) [file pone.0201946.s002.docx]

| **Short Motorcycle Riding Behavior Questionnaire(SMRBQ)** | | |
| --- | --- | --- |
| **Items in Persian** | **Item description in English** | **item** |
| **Main factor: Unfit erroneous riding, intrusive and exhibitive behaviors** | | |
| آنقدر نزدیک به وسیله نقلیه جلویی موتورسواری کنید که برای توقف در موقعیت­های اضطراری دچار مشکل شوید؟ | Tailgating the vehicles in front | 1 |
| برای گردش در پیچها، به طور باز گردش کرده و تمام عرض راه را اشغال کرده باشید؟ | Wide ride going round the corners | 2 |
| هنگام گردش در سر پیچ به دلیل سرعت بالا امکان داشته کنترل موتورسیکلت را از دست بدهید؟ | Speeding (when reaching corners) | 3 |
| آنقدر با سرعت وارد پیچ شوید، که خودتان دچار ترس شوید؟ | Scaring speeding (when reaching corners) | 4 |
| هنگام موتور سواری ترک چرخ بزنید یا قصد آن را داشته باشید؟ | Wheelie attempts | 5 |
| آنقدر پرگاز شروع به حرکت کنید که چرخ جلویی موتورسیکلت شما از مسیر جاده خارج شود؟ | Off road due to very quick pull away | 6 |
| به طور عمدی باعث چرخیدن چرخ­های موتورسیکلت در حالت در جا (تیک آف) شوید؟ | Wheel spin (on purpose) | 7 |
| به طور غیر عمدی باعث چرخیدن چرخهای موتورسیکلت در حالت در جا (تیک آف) شوید؟ | Wheel spin (unintentional) | 8 |
| در موقع تاریکی با چراغ خاموشموتورسواری کنید؟ | Riding at night just with dipped light | 9 |
| با موتورسیکلت معیوب رانندگی کنید؟ | Riding impaired motorbike | 10 |
| قبل از موتورسواری از دارو یا موادی که بر نحوه موتورسواری اثرگذار است، استفاده کرده باشید؟ | Riding while on drugs or medications affecting riding safety | 11 |
| خلاف جهت خیابان موتورسواری کرده باشید؟ | Riding against the legal traffic direction | 12 |
| در پیاده رو موتورسواری کرده باشید؟ | Sidewalk riding | 13 |
| در هنگام موتورسواری از موبایل استفاده کرده باشید(مکالمه یاپیامک)؟ | Mobile conversation or messaging while riding | 14 |
| **Second factor: Time and money opportunistic behaviors** | | |
|  | Speeding (motorways) | 15 |
|  |  |  |
| در خیابان­های موجود در اماکن مسکونی بیشتر از سرعت مجاز موتورسواری کنید؟ | Speeding(residential roads) | 16 |
| در فضای موجود بین دو باند سرعت رانندگی کنید؟ | Riding between fast lanes of traffic | 17 |
| با موتورسیکلت خود بار حجیم و بزرگ حمل کنید؟ | Carrying heavy weight | 18 |
| بر روی موتورسیکلت خود بیش از یک نفر ترک را سوار کنید؟ | Ride with more than one pillion passenger | 19 |
| دیر متوجه باز شدن درب وسیله نقلیه متوقف شده جلویی شده اید و به سختی از برخورد موتورسیکلت اجتناب کرده باشید؟ | Likely of hitting opened car doors | 20 |
| موقع عبور از تقاطع با وجود قرمز بودن چراغ راهنمایی، از تقاطع عبور کرده باشید؟ | Passing the red lights | 21 |
| **Third factor: Helmet use behaviors** | | |
| بدون کلاه ایمنیموتورسواری کنید؟ | Not using helmets while riding | 22 |
| فردی را بدون کلاه ایمنی بر روی ترک موتورسیکلت خود سوار کرده باشید؟ | Not using helmets by pillion passengers | 23 |
